# Oral Health Disparities Among Chinese Older Adults: Evidence From CHARLS

**DOI:** 10.1093/geroni/igaa057.262

**Published:** 2020-12-16

**Authors:** Chengming Han

**Affiliations:** Case Western Reserve University, Cleveland, Ohio, United States

## Abstract

This paper explored the effect of the type of health insurance on dentist visits among older adults in China. The data were drawn from the CHARLS-II (2013). The sample included older adults aged 60 and older (N= 6767, n(urban)=3272, n(rural)=3495). Multivariate logistic regression models indicated that in urban and rural places, respondents with a governmental/civil servants’ insurance and those with an urban-employee insurance are more likely to visit a dentist in the survey year. Household registration status (hukou) does not play a significant role in dentist visits when insurance types are adjusted for. In other words, employment status, and the coverage of health insurance presented more significant effects on dentist visits. Differing from previous studies about urban-rural health disparities, this study disclosed substantial institutional influences on dental care access among older adults.

